# Safety and Efficacy of 12-Month Intra-gastric Balloon—Series of over 1100 Patients

**DOI:** 10.1007/s11695-023-06953-0

**Published:** 2023-12-01

**Authors:** Tom Wiggins, Ojasvi Sharma, Yasmin Sarfaraz, Heather Fry, Julia Baker, Rishi Singhal

**Affiliations:** 1 Healthier Weight, Birmingham, UK; 2 University Hospitals Birmingham NHS Foundation Trust, Birmingham, UK; 3 Worcester Acute Hospitals, Worcester, UK; 4 Gastric Balloon Group, Birmingham, UK; 5 Birmingham City University, Birmingham, UK; 6 Gastric Balloon Group, Better Health Group, Birmingham City University, Birmingham, B15 3TN UK

**Keywords:** Bariatric surgery, Intra-gastric balloon, Weight loss, Safety, Efficacy

## Abstract

**Background:**

Intra-gastric balloons (IGB) are a mainstay of endoscopic treatment of overweight and obesity. In recent years, an IGB which can remain in situ for 12 months has been developed. The current study aimed to analyse the safety and efficacy of this 12-month IGB.

**Methods:**

Consecutive patients receiving the Orbera 365^TM^ IGB (Apollo Endosurgery, TX, USA) between September 2017 and August 2021 were included in a prospective database. Patients received regular follow-up consultations followed by endoscopic removal at 12 months. Demographic data along with weight loss data were collected. All adverse events were recorded.

**Results:**

In total, 1149 patients were included in the study. A majority of the patients were female (87.13%). Median body mass index (BMI) prior to insertion was 36.30 kg/m^2^ (IQR 32.60–40.00 kg/m^2^). Median absolute weight loss for all patients was 11.36 kg (IQR 6.70–16.82 kg). There was ongoing sustained weight loss until device removal at week 52. For patients with a weight recording at point of IGB removal, median weight loss was greater (15.88 kg, IQR 10.43–21.72) with percentage total body weight loss of 15.38% (IQR 10.99–21.77) and excess weight loss of 53.99% (IQR 32.44–76.30). Increased patient engagement with post-procedural follow-up was associated with increased weight loss (*p*<0.001). There were 60 total complications (5.22%). Fifty patients required balloon removal due to intolerance. There were eight cases of balloon rupture. There were only two severe complications (0.17%).

**Conclusion:**

The current study has confirmed safety of this IGB at 12 months with adverse events comparable to published literature. Weight loss increased up until the point of removal at 12 months.

**Graphical Abstract:**

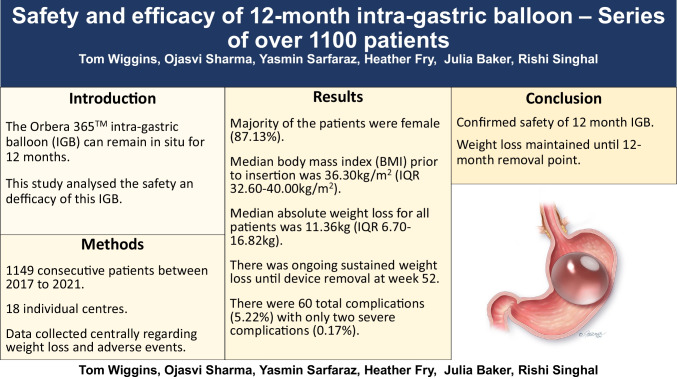

## Introduction

Overweight and obesity are rapidly increasing globally. Approximately 2 billion people worldwide are considered overweight and 650 million are suffering with obesity. Worldwide rates of obesity have tripled since 1975 [1]. In England, 68.6% of men and 59.0% of women are classified as suffering with overweight or obesity [2].

Metabolic and bariatric surgery (MBS) has long been established as the most effective treatment for patients with obesity to achieve long-term weight loss maintenance and resolution of co-morbidities [3, 4]. However, MBS is not suitable for all patients and less than 1% of eligible patients come forward to receive treatment [5]. Various endoscopic therapies for treatment of overweight and obesity have been developed. These procedures are capable of producing clinically meaningful weight loss, whilst being less invasive with comparable/lower peri-procedural risks as compared to MBS [6]. These procedures are therefore able to fulfil the treatment gap between MBS and conservative measures or pharmacotherapy for treatment of obesity and related co-morbidities.

Intra-gastric balloons were first developed around 1985 [7]. The technology regarding IGBs has developed significantly over time. Intra-gastric balloons are now the most widely utilised endoscopic therapy for treatment of overweight and obesity [8]. A recent meta-analysis has demonstrated that IGBs achieve significantly greater levels of total body and excess weight loss compared to conservative measures alone [9]. Sub-group analysis of the results from the Orbera™ balloon (Apollo Endosurgery, TX, USA) demonstrated significantly greater absolute weight loss compared to those receiving conservative treatment alone (7.99 kg difference in total weight loss (95% confidence interval (CI) 3.91–11.95)) [9].

The Orbera™ balloon was previously available to be inserted for a period of 6 months. This device has been demonstrated to achieve approximately 18% total body weight loss (TBWL) in a study of 5874 patients [10]. A new form of Orbera™ balloon has since become available which can be safely left in situ for up to 12 months rather than 6. The current study aimed to analyse the safety and efficacy of this 12-month Orbera 365™ IGB.

## Methods

All consecutive patients who received an intra-gastric balloon (IGB) using the Orbera 365™ device from a single private provider between September 2017 and August 2021 were included in this analysis. Data was collected prospectively on Salesforce (https://www.salesforce.com). The vast majority of patients had self-referred for treatment and met the World Health Organisation criteria for ‘overweight or obesity’ [1]. Patients were considered eligible for the procedure if they had a body mass index above 27 kg/m^2^ with or without obesity related co-morbidities. All endoscopists were accredited by the Joint Advisory Group on Gastrointestinal Endoscopy (JAG) [11] and received specific training regarding the technique of IGB insertion from Apollo Endosurgery (TX, USA).

**Fig. 1:**
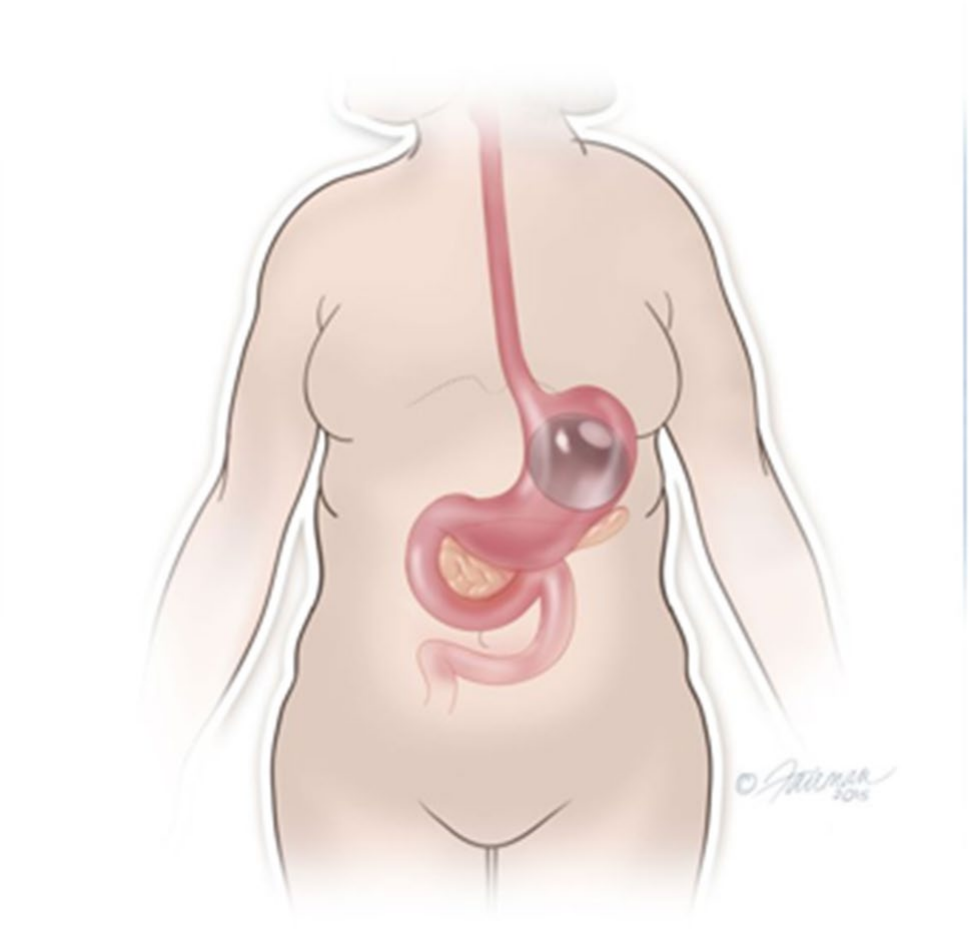
Graphical representation of IGB positioned within the stomach

**Fig. 2:**
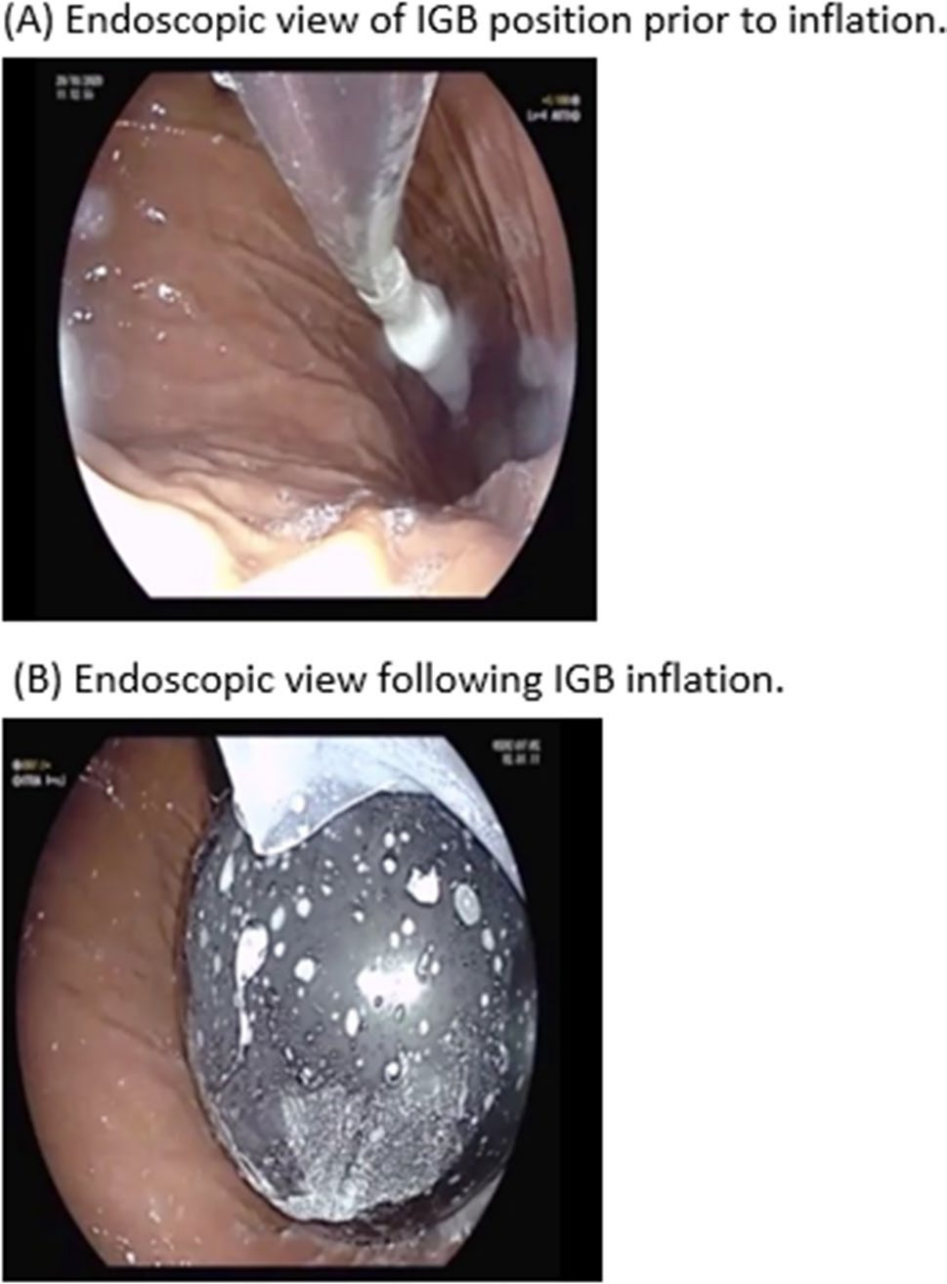
**A** Endoscopic view of IGB position prior to inflation. **B** Endoscopic view following IGB inflation

Prior to the procedure, patients were provided education regarding the IGB including details of the recommended dietary regimen following the procedure. Insertion of the Orbera 365™ IGB was performed endoscopically. This was performed either with or without sedation. Evaluation was undertaken to exclude a large hiatus hernia or other oesophago-gastric pathology which may preclude IGB insertion. The balloon was then positioned within the gastric fundus of the stomach and inflated under vision (with the endoscope in a retro-flexed position) using 450–600 ml saline or saline with methylene blue as per manufacturer instructions (based on practice of each endoscopist) (Figs. 1 and 2). All patients were given anti-emetic medications at the time of insertion. Towards the end of this data period, Akynzeo™ was utilised as a long-acting anti-emetic given at the time of insertion (gradual introduction from March 2021 onwards) [12]. Patients were asked to adhere to a dietary regimen which consisted of clear fluids for 2–3 days, followed by graduated progression from nutritious fluids and smooth purees to soft solids and normal diet.

Patients were then offered regular follow-up appointments to monitor weight followed by endoscopic removal 12 months following insertion.

### Data Analysis

Data collection included demographic and anthropometric data including age, sex, weight, comorbidities, body mass index (BMI), and short- and long-term morbidity and mortality. Weight outcomes were recorded at each follow-up time-point. Data was then anonymised and extracted for analysis using Microsoft Excel™ (Microsoft, NM, USA). Statistical analysis was performed using SPSS version 29 (IBM, NY, USA).

## Results

A total of 1149 patients who had the Orbera 365™ IGB inserted at 18 individual centres were included in the analysis. The median centre volume over the study period was 59 cases (inter-quartile range (IQR) 18–98.5).

### Patient Demographics

Patient demographics are included in Table 1. The vast majority of patients were female (87.13%). Most patients did not have any obesity-related co-morbidities. Only 2.13% of patients had diabetes at baseline, 5.89% had hypertension, and 1.80% had hypercholesterolaemia. Very few patients (0.49%) had obstructive sleep apnoea. Median weight prior to insertion was 101.46 kg (IQR 89.09–114.55 kg). This equated to a median body mass index (BMI) of 36.30 kg/m^2^ (IQR 32.60–40.00 kg/m^2^). Details of proportion of patients in each BMI category are provided in Table 1.

**Table 1:** Patient demographics

	Patient details (*n*=1149)
Median age (years)	40.67 (IQR 33.93–48.98)
Female sex	87.13% (968/1111)
Baseline diabetes	2.13% (13/611)
Baseline hypertension	5.89% (36/611)
Baseline hypercholesterolaemia	1.80% (11/611)
Baseline OSA	0.49% (3/611)
Baseline weight (kg)	101.36 (IQR 89.09–114.55)
Baseline body mass index (kg/m^2^)	36.30 (IQR 32.60–40.00)
Body mass index <30 kg/m^2^	95 (8.27%)
Body mass index 30–34.9 kg/m^2^	362 (31.51%)
Body mass index 35–39.9 kg/m^2^	382 (33.25%)
Body mass index 40–44.9 kg/m^2^	198 (17.23%)
Body mass index ≥ 45	111 (9.67%)

### Weight Loss Data

Following insertion, the median time prior to removal was 364 days (IQR 274–388 days) (Table 2). Follow-up weight data was available for 954 patients (83.03%). Median absolute weight loss was 11.36 kg (IQR 6.70–16.82 kg). This equated to 11.11% total body weight loss (TBWL) (IQR 6.67–16.45%) or 36.58% excess BMI loss (IQR 21.05–56.50%) (Table 3). Of the entire cohort with available weight loss data, 540 patients (56.60%) achieved 10% or greater TBWL, 255 patients (26.73%) achieved TBWL between 5 and 10%, and 159 (16.65%) achieved less than 5% TBWL.

**Table 2:** Procedure details and complications

Balloon in situ time	
Median in situ time (days)	364 (IQR 274–388)
Median in situ time (months)	11.96 (IQR 9.00–12.75)
Complications	
Total complications	5.22% (60/1149)
Early balloon removal	4.35% (50/1149)
Balloon rupture	0.70% (8/1149)
Gastric outlet obstruction	0.09% (1/1149)
Gastric perforation	0.09% (1/1149)

**Table 3:** Follow-up and weight loss data

Follow-up	
Follow-up weight available	83.03% (954/1149)
Weight loss outcomes (all patients)	
Weight loss (kg)	11.36 (IQR 6.70–16.82)
Total body weight loss (%)	11.11% (IQR 6.67–16.45)
BMI loss (kg/m^2^)	4.07 (IQR 2.41–6.13)
Excess BMI loss (%)	36.58% (IQR 21.05–56.50)
Weight loss outcomes (recorded at removal time) (*n*=211)	
Weight loss (kg)	15.88 (!QR 10.43–21.72)
Total body weight loss (%)	15.38% (10.99–21.77)
BMI loss (kg/m^2^)	5.66 (3.68–8.02)
Excess BMI loss (%)	53.99 (IQR 32.44–76.30)

For patients with recorded weights specifically around the point of balloon removal (defined as between weeks 44 and 52), there was greater percentage TBWL (15.38%) (IQR 10.99–21.77), and excess % weight loss (53.99%) (IQR 32.44–76.30) (Table 2).

**Fig. 3:**
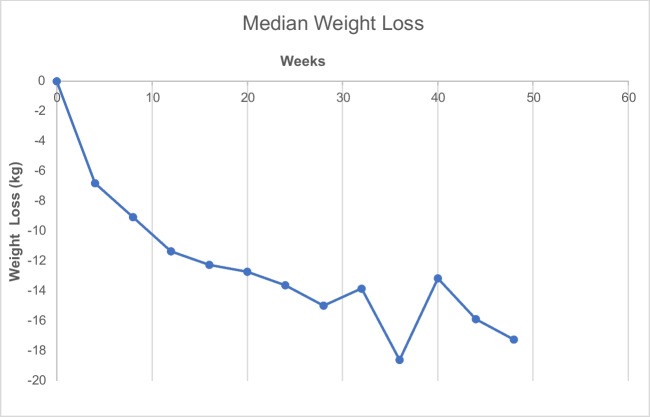
Median weight loss (kg) over time

**Fig. 4:**
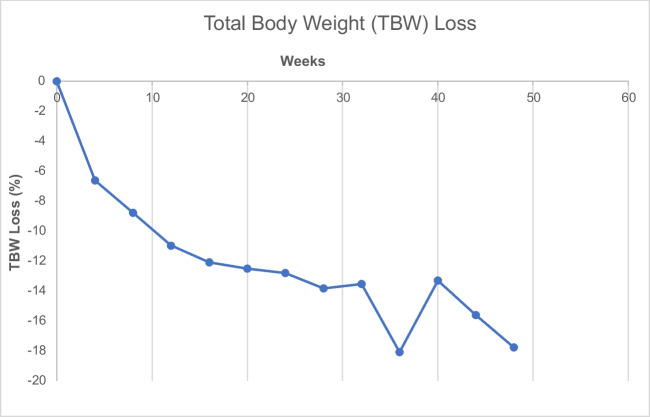
Median total body weight loss (%) over time

Interval weight loss over time is provided in Fig. 3. This demonstrates initial rapid weight loss in the first 12 weeks following IGB insertion. Weight loss then continued at a slower rate until device removal. Although peak weight loss in the present data was achieved at week 36, the general trend identified in the present analysis was ongoing sustained weight loss until device removal at week 52. A similar trend was identified for percentage TBWL (Fig. 4).

Increased engagement with post-procedural follow-up appointments appeared to be associated with increased weight loss. Patients who attended one or fewer follow-up appointments with the specialist team achieved a median TBWL of 8.07% (IQR 4.74–12.97). Those who attended two to three appointments achieved a median TBWL of 11.86% (IQR 7.39–16.70) and those attended four or more appointments achieved median TBWL of 14.17% (IQR 9.54–19.80) (Krukal-Wallis test *p*<0.001).

Weight loss by BMI category is provided in Table 4. As anticipated, the highest absolute weight loss was achieved in patients with BMI ≥ 45 (13.61 kg, IQR 6.35–20.19) (Kruskal-Wallis test c across all groups). Excess % weight loss was greatest within the lower BMI groups (BMI <30 kg/m^2^, 78.28% (IQR 45.24–124.47) vs. BMI > 45 kg/m^2^, 22.41% (IQR 4.92–15.42) (*p*<0.001)). Highest TBWL was achieved in patient with BMI 35–39.9 kg/m^2^ (11.76% (IQR 7.55–16.96%)) and BMI 30–34.9 kg/m^2^ (11.57% (IQR 6.94–16.31%)) groups but there was no significant difference in percentage TBWL across all groups (Kruskal-Wallis test *p*<0.185).

**Table 4:** Weight loss by BMI category

BMI <30 kg/m^2^ (*n*=95)	
Weight loss (kg)	8.18 (IQR 4.43–11.93)
Total body weight loss (%)	9.99% (IQR 5.92–15.38)
BMI loss (kg/m^2^)	3.03 (IQR 1.59–4.14)
Excess BMI loss (%)	78.28% (IQR 45.24–124.37)
BMI 30–34.9 kg/m^2^ (*n*=362)	
Weight loss (kg)	10.00 (IQR 6.36–15.00)
Total body weight loss (%)	11.57% (IQR 6.94–16.31)
BMI loss (kg/m^2^)	3.61 (IQR 2.04–5.41)
Excess BMI loss (%)	49.92% (IQR 29.47–71.49)
BMI 35–39.9 kg/m^2^ (*n*=382)	
Weight loss (kg)	12.27 (IQR 7.73–17.73)
Total body weight loss (%)	11.76% (IQR 7.55–16.96)
BMI loss (kg/m^2^)	4.38 (IQR 2.67–6.37)
Excess BMI loss (%)	35.84% (IQR 23.32–52.27)
BMI 40–44.9 kg/m^2^ (*n*=198)	
Weight loss (kg)	12.25 (IQR 7.15–18.60)
Total body weight loss (%)	10.33% (IQR 6.19–16.28)
BMI loss (kg/m^2^)	4.44 (IQR 2.71–6.80)
Excess BMI loss (%)	27.31% (IQR 15.82–40.10)
BMI ≥ 45 kg/m^2^ (*n*=111)	
Weight loss (kg)	13.61 (IQR 6.35–20.19)
Total body weight loss (%)	10.31% (4.92–15.42)
BMI loss (kg/m^2^)	4.88 (2.67–7.49)
Excess BMI loss (%)	22.41% (4.92–15.42)

### Early Removals and Complications

Details of procedural complications are provided in Table 2. A total of 60 patients (5.22%) had a complication. Fifty patients (4.35%) required early balloon removal due to intolerance. Twelve patients had the balloon removed within 7 days of insertion and the other 38 patients had the balloon removed between 8 and 38 days after insertion. Of the group of patients who received Akynzeo™ (*n*=132), there were five patients who required early device removal (3.79%). There was no significant difference between this and the rate of early balloon removal for patients who did not receive Akynzeo™ (*n*=45/1017, 4.42%) (Chi-squared *p*=0.736).

There were eight cases of balloon rupture (0.70%) which all passed spontaneously. There were two severe complications (overall rate of severe complications 0.17%). There was one case of gastric outlet obstruction which resolved following conservative treatment . There was one case of gastric perforation which required laparotomy for repair .

## Discussion

The current study represents the largest series regarding weight loss outcomes with the Orbera 365™ balloon and demonstrates the clinical efficacy and safety of the procedure. Overall median TBWL for all patients was 11.11% (IQR 6.67–16.45) with a 36.58% excess BMI loss (IQR 21.05–56.50%). Patients with increased engagement with follow-up attendances achieved significantly greater weight loss (median TBWL if four or greater follow-up attendances 14.17% compared to 11.86% with two to three visits and 8.07% with zero to one visit (*p*<0.001)). Rate of early balloon removal was acceptable (4.35%) and rate of major complications was low.

The TBWL achieved for patients at around 12 months post-insertion appears comparable to the available data regarding weight loss with the previous form of Orbera™ balloon which was typically removed after around 6 months [10]. Fittipaldi-Fernandez et al. reported outcomes from a larger group of patients utilising the 6-month Orbera™ balloon and demonstrated TBWL of 18.42 ± 7.25% [10]. This appears to be similar to the data at 12 months for patients presented in the current study (median TBWL 15.38% (IQR 10.99–21.77)). Weight loss at the 6-month time point appears to have been greater in the study by Fittipaldi-Fernandez et al. study and the reasons for this are unclear as patient demographics and proportion of patients in each BMI category were similar. Despite this, the current intervention still met the criteria for endoscopic bariatric therapies stated by the American Society of Gastrointestinal Endoscopy (ASGE) and American Society of Metabolic and Bariatric Surgery (ASMBS) of achieving excess weight loss at least 15% greater than conservative therapies [13]. A previous meta-analysis has demonstrated that IGBs appear to achieve 17.98% additional excess weight loss and 4.40% total body weight loss relative to conservative treatments [9]. This meta-analysis had utilised data from randomised trials investigating all forms of IGB but identified particular significance of the effect of the Orbera™ IGB upon results [9].

Although TBWL appears to have been similar with the 12-month Orbera 365™ IGB compared to the previous 6-month version, it is important to note that this demonstrates that weight loss is maintained beyond the 6-month time point. Previous iterations of IGB have been troubled with weight regain following device removal [14, 15]. Although details regarding weight change following device removal were not available in the present study, the fact that the Orbera 365™ IGB is able to maintain weight loss for a longer period may provide greater long-term weight loss with reduced weight regain following device removal. This will be an area that requires further investigation in future studies.

Rates of major complications in the current study were low. There were only two major complications (one gastric outlet obstruction and one gastric perforation) with an overall rate of major complications of 0.17%. There were 58 other patients who required early balloon removal or suffered balloon rupture to provide an overall complication rate of 5.22%. This rate is lower than that reported from an analysis of the Metabolic and Bariatric Surgery Accreditation and Quality Improvement Program (MBSAQIP) which reported data from 2910 IGB patients and identified a complication rate of 9.9% [8]. The current results are similar to those identified by Fittipaldi-Fernandez et al. who had reported an overall complication rate of 7.32% with the majority of these relating to early device removal (6.10%) [10].

With specific regard to these patients who required early balloon removal (50/1149 (4.35%)), the use of Akynzeo™ was added towards the end of the current study period as a peri-operative long-acting antiemetic. This had been provided to 132 patients and these individuals had an early removal rate of 3.79% which was not significantly less than that for the other cohort of patients who received traditional anti-emetic treatment (4.42%) (*p*=0736). However, the current cohort does not represent a large enough study group to draw firm conclusions regarding the benefits of Akynzeo™ in IGB insertion and this should be an area for further additional study as currently there are no other large-scale comparative studies between Akynzeo™ and standard anti-emetic therapy for IGB insertion [12].

In relation to outcomes in the different classes of obesity, absolute weight loss demonstrated increasing quantity of weight loss across the increased BMI thresholds (BMI <30 kg/m^2^, 8.18 kg (IQR 4.43–11.93) vs BMI ≥45 kg/m^2^, 13.61 kg (IQR 6.35–20.19) (*p*<0.001)). However, the patients within the lower BMI groups had significantly greater excess % weight loss compared to the higher groups (BMI <30 kg/m^2^, 78.28% (IQR 45.24–124.37) vs BMI ≥ 45 kg/m^2^, 22.41% (IQR 4.92–15.42) (*p*<0.001)). These results are again similar to those identified by Fittipaldi-Fernandez et al. using the 6-month Orbera™ balloon [10].

Previous studies have demonstrated that although IGBs may achieve short-term weight loss, there can be issues regarding long-term weight regain [15]. However, it is important to consider that MBS is not suitable for all patients and some may not be willing to accept this form of invasive procedure. Alongside being less invasive, IGBs carry the additional benefit of being entirely reversible which can make them a more acceptable treatment option for some individuals. With the advances in pharmacological therapies for treatment of obesity in recent years, it will be critical to develop a multi-modality strategy for management of obesity incorporating pharmacological, endoscopic, and surgical therapies to provide an individualised patient-centred approach to achieve the desired outcome. It is anticipated that healthcare professionals would be able to discuss all the potential treatment options (IGB, MBS, or pharmacological treatment) and enable the patient to make an informed decision regarding which treatment best suits their individual characteristics and desired outcome. For some patients, use of IGB alone will provide a potentially effective treatment which can help them to achieve their individual weight loss targets whilst maintaining the option for alternative procedures at a later point in their treatment. In addition, there is evidence that even a short period of weight normalisation can have a long-term impact upon metabolic outcomes [16]. Although there is evidence that some pharmacological therapies can achieve weight loss outcomes similar to those identified with IGB insertion [17], it is important to note that some patients are unable to tolerate these treatments (largely due to gastrointestinal disturbance as a significant side effect) or do not wish to administer regular injections for those treatments where this is necessary. This will likely mean that some patients would still consider IGB placement as their preferred option for treatment of obesity.

There are important limitations which must be considered when interpreting the results presented here. Weight loss data was recorded in a central database following each patient contact directly within our service. Unfortunately as balloons were inserted and removed endoscopically at independent treatment centres, the record of patient weight at the point of removal was not available for all patients and this is a limitation of the current study. However, every effort was made to ensure this dataset was as complete as possible. It is also true that weight loss data for week 36 of follow-up appeared to be greater than anticipated and then returned to the expected trend by week 40. There was no identifiable reason for this and hence this was included within the presented results. No data was collected regarding resolution of co-morbidities during the period of the balloon being in situ; and therefore, the authors were unable to comment on any changes in co-morbidity status over the study period. An additional limitation is that no data was collected regarding patient weight change following device removal; and therefore, it is not possible to draw conclusions regarding long-term weight outcomes (beyond 12 months) from the current dataset.

Future research in this area should aim to establish if the maintenance of weight loss over the longer period of 12 months leads to better long-term weight maintenance following device removal compared to 6-month IGBs. It is hoped that the longer period of weight loss would assist in avoiding weight regain which has been an issue with shorter term balloons. It will also be important for further research to define the role of IGB placement alongside the other available modalities for treatment of obesity (including MBS and pharmacological therapies). It is hoped that IGB can be utilised alongside these other therapeutic options to provide a treatment pathway tailored to the individual patient.

## Conclusion

In conclusion, the current study represents the first report of large-scale results using the Orbera 365™ balloon. This has demonstrated that utilisation of a 12-month IGB can achieve meaningful weight loss with an acceptable risk profile. Future areas for research will help to define the role of IGB placement alongside other available treatment modalities (including MBS and pharmacological treatments of obesity) to facilitate an individualised management strategy for each patient seeking treatment for obesity.

## Data Availability

Data can be made available upon receipt of the relevant permisssions.
